# Raman Spectroscopy Investigations of Ribbeck Meteorite

**DOI:** 10.3390/ma17205105

**Published:** 2024-10-19

**Authors:** Mariusz Dudek, Jacek Grabarczyk, Tomasz Jakubowski, Paweł Zaręba, Anna Karczemska

**Affiliations:** 1 Institute of Materials Science, Lodz University of Technology, Stefanowskiego 1/15, 90-537 Lodz, Poland; mariusz.dudek@p.lodz.pl (M.D.); jacek.grabarczyk@p.lodz.pl (J.G.); 2 Polish Meteorite Society, Bedzinska 60, 41-200 Sosnowiec, Poland; illaenus@gmail.com (T.J.); meteorite@o2.pl (P.Z.); 3 Institute of Turbomachinery, Lodz University of Technology, Wolczanska 217/221, 93-005 Lodz, Poland

**Keywords:** meteorite, aubrite, Raman spectroscopy, SEM EDS

## Abstract

On 21 January 2024, asteroid 2024BX1, discovered the three hours before, fell to Earth south of Ribbeck in the Havelland region of Germany. In this study, fragments of the Ribbeck meteorite, characterized by white and gray colors lithology, were examined for their chemical and phase compositions. The white lithology fragment exhibited a homogeneous chemical and phase structure typical of orthopyroxene, which crystallizes in the orthorhombic system. The gray lithology fragment showed a greater diversity in chemical and phase compositions. Raman spectra analysis revealed that, in addition to the pyroxenes found in the white lithology fragment, minerals from the olivine group (fayalite and forsterite) were also present, along with plagioclase and sulfur in pure crystalline form.

## 1. Introduction

Meteorites are rocks that originate in outer space and have fallen to the surface of the Earth. They have fascinated humans for thousands of years, even before we could understand their origin. It is interesting to note that the first iron used by humans came from meteorites. Studying meteorites currently enhances our understanding of the solar system and the universe. Similarly, just as it did thousands of years ago, it also drives innovation in materials science and technology. Modern materials science techniques allow us to investigate these samples of materials manufactured in outer space under very different conditions from those known on Earth.

On 20 January 2024 at 21:48 UTC, Hungarian astronomer Krisztián Sárneczky discovered asteroid 2024BX1 [1]. The bolide was also recorded by the European Fireball Network, IMO/All-Sky7, and the Fireball Recovery and Inter-Planetary Observation Network (FRIPON). A meteoroid, 1 m in size and 140 kg in mass, was predicted to enter the Earth’s atmosphere three hours before its fall [2]. The impact was predicted to occur west of Berlin, in the Havelland region of Germany. The meteorite fell on 21 January, south of Ribbeck. The first Ribbeck meteorite was found on 25 January: a single stone weighing 171 g, broken into three pieces, was found by Polish hunters. The main mass, weighing 225 g, was found the next day, also by a Polish hunter. About 200 pieces, with a total mass of approximately 1.8 kg, were recovered in a short period of time [3].

Three weeks after the fall, the Ribbeck meteorite was classified by Dr Ansgar Greshake from the Natural History Museum in Berlin as an aubrite (Meteoritical Bulletin Database). It is described as a heavily brecciated aubrite consisting of 76 ± 3 vol % FeO-free enstatite, 15 ± 2.5 vol % albitic plagioclase, 5.5 ± 1.5 vol % forsterite, and 3.5 ± 1.0 vol % sulfides and metals [4]. Ribbeck-2024BX1 is the eighth asteroid in contemporary history discovered before impacting Earth and only the twelfth recorded observed fall of an aubrite in history [1,4]. Most aubrites are found in cold and hot deserts, with 77 different finds officially listed in the Meteoritical Bulletin.

Aubrites are a rare type of enstatite achondrite meteorite with a unique mineralogical composition. Based on oxygen isotopes, they can be related to enstatite chondrites [5]. They are mostly composed of Fe-free enstatite (a mineral high in magnesium and low in silicon), minor forsterite, albite, and rare minerals such as sulfides, nitrides, and carbides [6]. The formation age of aubrites has been measured from 4560 Ma to 4550 Ma, suggesting two distinct groups of aubrites [5]. Aubrites are most likely related to E-type asteroids, where the spectra of meteorites and asteroids are similar [5]. Bischoff et al. [4] indicate that the chemical composition of Ribbeck meteorite is close to the composition of other aubrites, for instance Bishopville, Aubres or Norton County. They also indicate that Ribbeck is not a regolith breccia, but a fragmental breccia. The brecciated texture of Ribbeck clearly indicates the significant impact activities that have affected its parent body. Additional impact events are necessary to account for the formation of small shock-melted areas and shock veins within Ribbeck’s fine-grained clastic matrix.

In recent decades, Raman spectroscopy has gained importance in the study of meteorites [7,8,9,10,11,12,13,14,15,16,17,18,19,20,21,22,23,24,25]. This technique allows non-destructive analysis of the mineral composition of the meteorite samples. It is highly sensitive to different minerals and provides the information about molecular structure and chemical bonds in the investigated materials. It can also detect both crystalline and amorphous materials, it shows the differences in the composition of different points of the same mineral, and it also allows for an understanding of the distribution of different minerals in the sample. Additionally, the studies using Raman spectroscopy can be performed on small sample sizes and special surface preparation is not required, so the technique is even less invasive.

**Figure 1:**
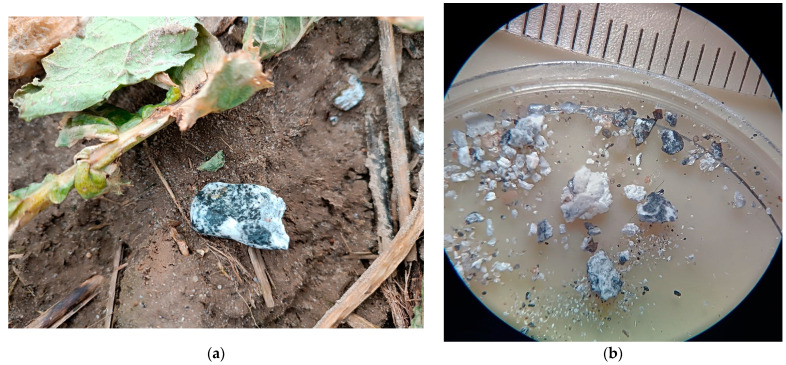
Ribbeck meteorite found by Paweł Zaręba on 2 February 2024: (**a**) meteorite photo as it was found—9.2 g; (**b**) meteorite crumbs used for studies in this article.

This paper aims to characterize the chemical and phase compositions of the Ribbeck meteorite shown in Figure 1. The white and gray fragments were examined using a scanning electron microscope (SEM) with energy dispersive spectroscopy (EDS) and Raman spectrometry. These studies allowed us to identify the minerals that compose the Ribbeck meteorite. In relation to the structure of previously identified meteorites, this will allow for a better understanding of the structure of the solar system. Information about the chemical and phase compositions will inspire the search for new materials for their applications in everyday use.

## 2. Materials and Methods

**Figure 2:**
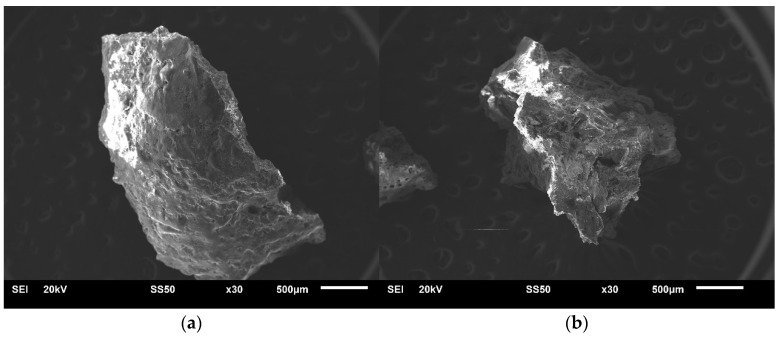
SEM images of two Ribbeck meteorite slivers selected for study, macroscopically characterized by (**a**) white color lithology; (**b**) gray color lithology.

Fragments of the Ribbeck meteorite (Figure 1b) described in the introduction were examined for structure and chemical composition with the use of a scanning electron microscope JSM-6610LV (JEOL) integrated with EDS X-MAX 80 (Oxford Instruments, Abingdon, UK) analyzer. To document the sizes of the examined irregular meteorite slivers, SEM images were taken at 30× magnification (Figure 2). EDS analysis of the chemical composition was performed for flat areas at 300× magnification (the map covered an area of 1.4 × 10^5^ μm^2^).

The chemical structure was also investigated using Raman scattering spectroscopy. The research was carried out by the Renishaw inVia Raman Microscope equipped with a 532 nm laser arranged in a backscattering geometry. The investigated wavenumber ranged from 100 to 3200 cm^−1^. The measurements were carried out using two objectives with a magnification of 5× and 50×, corresponding to a spot size of 10 and 1 μm in diameter, respectively. The remaining parameters (laser power, exposure time and accumulation) were selected to obtain good-quality spectra. All measurements were carried out in air at room temperature. After bassline subtraction, the Raman spectra were analyzed qualitatively.

## 3. Results and Discussion

After the initial macroscopic assessment of the meteorite, two slivers characterized by white and gray colors lithology (Figure 1b) were selected for research using the EDS technique coupled with SEM and a Raman spectrometer. Figure 2 shows their photos taken using SEM. The chemical composition of these slivers was then examined using the EDS technique, with the measurement results (average values from the areas for which the maps were made) presented in Table 1. For the white sliver, the chemical composition maps indicate homogeneity. In contrast, for the gray sliver the analyzed areas (maps) differed in chemical composition, showing regions with increased concentrations of certain elements. It should be emphasized that the results show a lack of sodium in the chemical composition analyses of the gray sliver, whereas in the white sliver, sodium is present on the surface at a level of ~1.4 at.%.

The results of the EDS analysis (Table 1) show that the most common element is oxygen, which suggests that the meteorite in question consists of silicon oxides and metals such as Mg, Al, Cr, Fe, Co, Ti, Ni, and Mo. By relating this local information on the chemical composition to the chemical composition of larger fragments of the Ribbeck meteorite [4], it can be seen that the results presented in Table 1 well reflect the overall chemical composition. It is worth noting that the local analysis of the chemical composition shows that the high sulfur content (3.09 at. %) is accompanied by a high iron content (9.87 at. %) at the expense of the presence of silicon and other metals (in particular, the absence of aluminum). This indicates that the gray slivers of meteorite will also be characterized by a large diversity in terms of phase composition.

**Figure 3:**
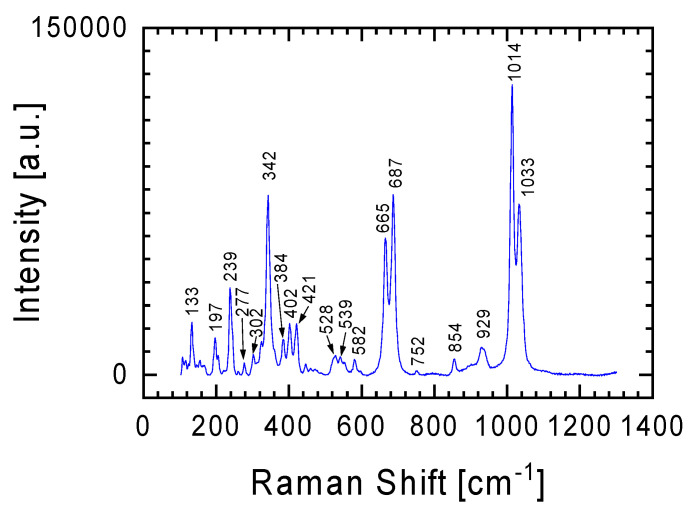
An example of a Raman spectrum obtained for a white lithology meteorite sliver characterized by a homogeneous chemical composition. Spectrum obtained using an objective with 5× magnification.

In order to determine the phase composition of these slivers, tests were conducted using a Raman spectrometer. Two lenses, 5× and 50× magnification, were used for the tests. The Raman spectra of the white sliver looked almost identical at randomly selected study points, regardless of the lens used. Figure 3 illustrates a characteristic spectrum of the sliver, showing Raman lines at 342 cm^−1^ (M-O stretch), 665 cm^−1^ (Si-O-Si stretch), 687 cm^−1^ (Si-O-Si stretch), 1014 cm^−1^ (Si-O bridging stretch), and 1033 cm^−1^ (Si-O bridging stretch), which are characteristic of the orthopyroxene enstatite [7,8,9].

The EDS analysis of the meteorite slivers (Table 1) in reference to the general formula of pyroxenes (XY(Si,Al)_2_O_6_, where X represents Ca, Na, Fe(II), or Mg, and Y represents ions of smaller size, such as Cr, Al, Mg, Co, Ti) confirms this assumption. The Raman spectra of orthopyroxene are very similar to the spectra of clinopyroxene (pyroxenes that crystallize in the monoclinic system). Therefore, it can be assumed that a small contribution of the clinopyroxene phase may also be present in the spectra [10,11,12,13].

**Figure 4:**
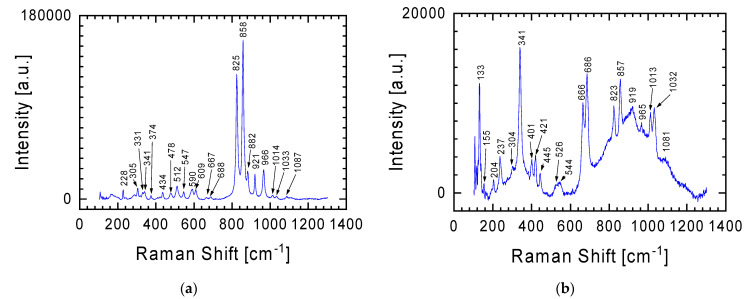
Examples of two subsequent Raman spectra, next to the spectrum in Figure 3, were recorded for a gray lithology sliver characterized by greater chemical diversity in individual places on the surface. Spectrum obtained using 5× magnification objective.

In the case of the gray lithology meteorite fragment, characterized by greater chemical diversity in individual places (see Table 1), in addition to the spectrum already presented in Figure 3 for the white lithology fragment, other spectra were recorded, two representative examples of which are presented in Figure 4. The characteristic Raman lines at 825 and 858 cm^−1^ in Figure 4a are fingerprints of olivine with the chemical formula (Mg,Fe)_2_SiO_4_ [14,15,16]. The lines are assigned to the Si-O antisymmetric and symmetric stretching vibration of the SiO_4_ group (825 and 858 cm^−1^, respectively). It should be emphasized here that Raman spectra also allow the differentiation of minerals in the olivine group. From the perspective of the Raman lines presented in Figure 4, attention should be paid to two members of this group: (i) fayalite (Fe_2_SiO_4_, black), the iron-rich end-member, and (ii) forsterite (Mg_2_SiO_4_, light yellow-green), the magnesium-rich end-member of the olivine solid-solution series [17]. In the case of the spectrum in Figure 4, it can be said that both of these minerals are present in the tested sample. The higher intensity of the Raman line at 858 cm^−1^ than at 825 cm^−1^ indicates that the dominant contribution to the spectrum comes from forsterite. This is also evidenced by the presence in the Raman spectrum of lines at 228, 305, 434, 590, 609, 882 and 966 cm^−1^, characteristic of magnesium-rich end-member of the olivine solid-solution series. However, Raman lines at 374, 547, and 921 cm^−1^ indicate the presence of fayalite.

In the Raman spectrum from Figure 4a, one can also distinguish lines at 478 and 512 cm^−1^, discussed in detail when discussing below the spectrum from Figure 7 (obtained using an objective with 50× magnification), which should be assigned to plagioclase [13,18,19].

The Raman spectrum in Figure 4b is a specific combination of the spectra from Figure 3 and Figure 4a, with the Raman line at 341 cm^−1^ becoming more prominent. The Raman line characteristic of orthopyroxene was previously discussed in relation to the spectrum from Figure 3. Additionally, this spectrum (Figure 4b) may also suggest an initiated transformation at a moderate shock level of plagioclase to maskelynite. This is indicated by a broad peak with a maximum at 919 cm^−1^, which contains characteristic bands for orthopyroxene and plagioclase [20,21]. It should also be emphasized that this spectrum was obtained using a 5× magnification objective. Thus, the signal comes from a relatively large area in which a large number of different forms are characterized by short-range order (broad band between 600 and 1200 cm^−1^), against which lines from forms with greater order can be seen. The confirmation that the examined place of meteorite could be described as quasi-amorphous is also evidenced by the low intensity of the lines outside this area of spectrum compared with the spectra presented in other figures.

**Figure 5:**
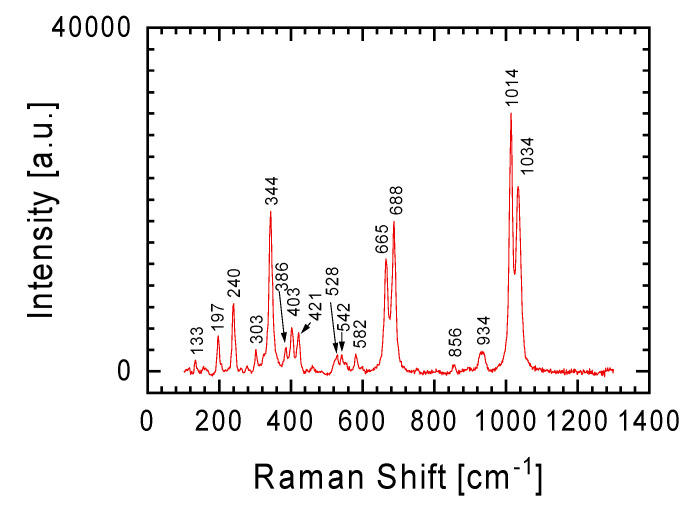
Raman spectrum of orthopyroxene obtained for a white lithology meteorite sliver using an objective with 50× magnification. This spectrum is identical to the spectrum obtained with the 5× objective shown in Figure 3.

In order to separate the spectra coming from different minerals in the tested sample, tests using a Raman spectrometer were also carried out using a 50× magnification objective. The homogeneity of the chemical composition for the white lithology meteorite sliver (Table 1) was confirmed by these results. As was the case with measurements with the 5× objective (Figure 3), measurements with the 50× objective gave the same spectrum (Figure 5) regardless of the location chosen on the sample for testing. In the case of the gray lithology meteorite sliver, measurements with the 50× objective allowed for the isolation of a larger number of minerals constituting this sliver than in the cases of Raman spectra obtained from measurements with the 5× objective presented above.

**Figure 6:**
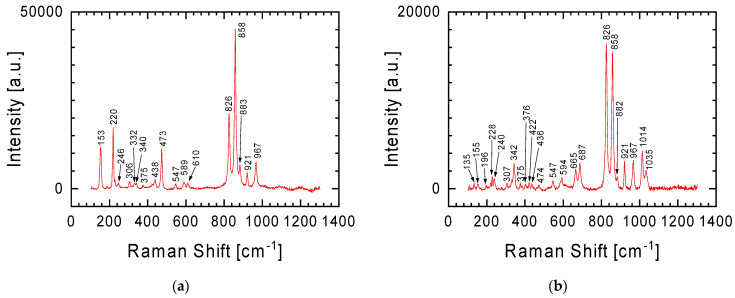
Raman spectra of minerals in the olivine group obtained for a gray lithology meteorite sliver using a 50× objective: (**a**) forsterite-like; (**b**) fayalite-like.

The research results presented above show that olivine is one of the minerals identified in the gray lithology meteorite fragment. The analysis of the spectrum in Figure 4a clearly shows that in the studied meteorite, forsterite and fayalite can be distinguished within the olivine groups. Figure 6 with spectra obtained at 50× objective magnification allows for a clear distinction between these two elements of the olivine group. Referring to the chemical composition of this meteorite fragment (Table 1), for places richer in magnesium we have spectra corresponding to forsterite with Raman lines at 220, 306, 438, 589, 610, 826, 858, 883, and 967 cm^−1^ (Figure 6a), and for those richer in iron, spectra correspond to fayalite from Raman lines at 240, 375, 547, 826, 858, and 921 cm^−1^ (Figure 6b). Importantly, in the spectrum assigned to forsterite, the intensity of the Raman line at 858 cm^−1^ is higher than that of the Raman line at 826 cm^−1^, while in the spectrum assigned to fayalite, Raman lines considered characteristic of olivine have almost identical intensity. Nevertheless, in both spectra (Figure 6) we can find Raman lines, which should be attributed to second of the considered elements of the olivine group. This means that both forms of the mineral contribute to the individual meteorite crystallites. When examining the chemical composition of the gray meteorite fragment, we see that magnesium and iron are present in every examined place, but their amount is not constant and depends on the place (Table 1).

Looking at the chemical composition of the gray lithology meteorite fragment (Table 1), we see that calcium is an important element. This means that in the tested sample we can also expect elements of the olivine group such as monticellite (CaMgSiO_4_) and kirschsteinite (CaFeSiO_4_), and gray silicate minerals [19]. Unfortunately, it is difficult to clearly distinguish the Raman lines of these minerals from the presented spectra (Figure 6) due to the high similarity to the above-discussed elements of the olivine group (forsterite and fayalite).

The difficulty in separating other elements of the olivine group in the spectra presented in Figure 6, apart from forsterite and fayalite, does not mean that these spectra are devoid of inclusions from the minerals already discussed. Similarly to the spectrum from Figure 4b (objective magnification 5×), in the spectrum from Figure 6b we can distinguish Raman lines typical for orthopyroxene: Raman lines at 342, 665, 687, 1014 and 1035 cm^−1^.

**Figure 7:**
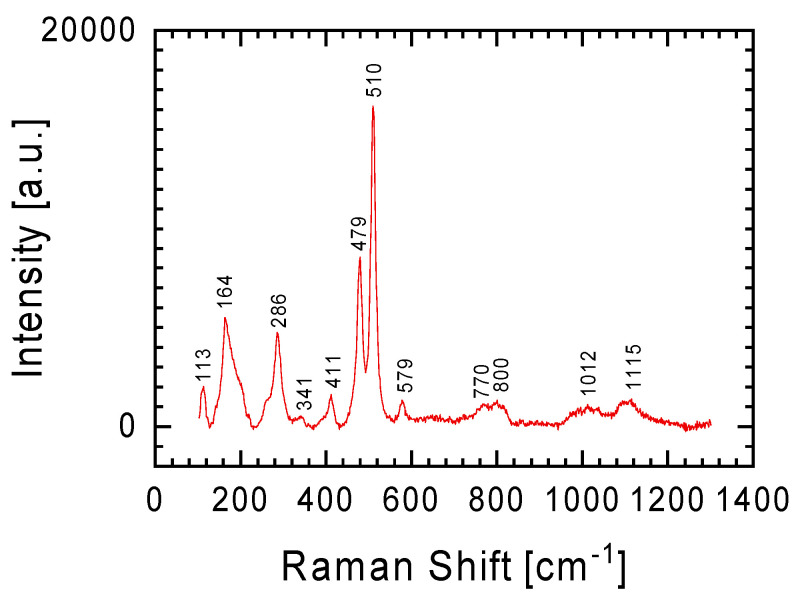
Raman spectrum of plagioclase obtained for the gray lithology fragment of the Ribbeck meteorite. Spectrum obtained using an objective with 50× magnification.

The less intense Raman lines visible in Figure 4a are the lines at 478 and 512 cm^−1^. These bands with a slightly shifted position (lines at 479 and 510 cm^−1^, respectively) are clearly visible in the spectrum obtained at 50× objective magnification (Figure 7). These lines (Si(Al)-O-Si(Al) bend), as well as the weak line at 286 cm^−1^ (Si-O-Si bend), should be assigned to plagioclase, a solid solution of the albite Ab (NaAlSi_3_O_8_) and anorthite An (CaAl_2_Si_2_O_8_) minerals [13,18,19]. The lack of Na in the examined meteorite fragment (Table 1) indicates that in our case only anorthite contributes to the spectrum in Figure 7. It is worth mentioning here that in the parallel studies on the Ribbeck meteorite by Bischoff et al. [4], lines at 284, 326, 463, and 500 cm^−1^ were also identified in the Raman spectra, and in the presence of potassium in its chemical composition these lines were considered as characteristic of the K-feldspar-like phase (KAlSi_3_O_8_). In the case of the discussed meteorite fragments, potassium was not identified (Table 1).

**Figure 8:**
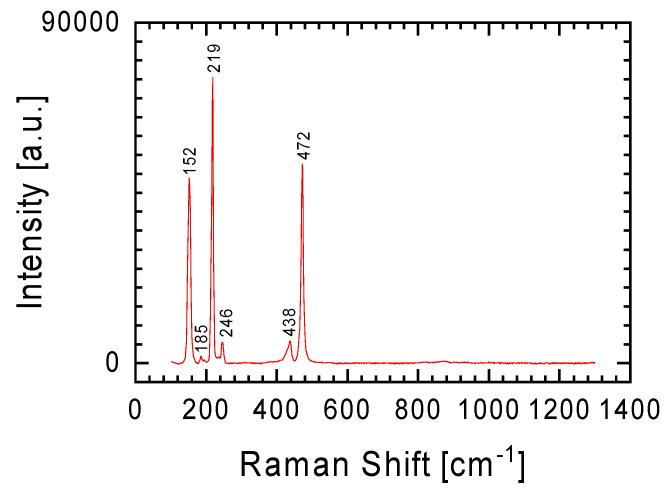
Raman spectrum of sulfur crystallite present in a gray lithology meteorite fragment. Spectrum obtained using an objective with 50× magnification.

In the introduction, it was mentioned that the meteorite was found shortly after it fell in January 2024. The finders reported that the found meteorites smelled of sulfur. The tested meteorite fragments showed that a gray lithology fragment may contain up to 3.1 at.% sulfur. It is uncertain whether the sulfur exists in its elemental state or as an oxide, as mentioned by Bischoff et al. [4]. The Raman spectrum in Figure 8 displays lines at 152, 219, 246, 438, and 472 cm^−1^, indicating the presence of sulfur crystals in the meteorite (lines belonging to stretching vibration of S=S molecules) [26]. This finding led to a reevaluation of the discussed spectra, suggesting that the presence of sulfur in the meteorite is the source of the less intense Raman lines in the spectra from Figure 4a and Figure 6. For example, lines at 155, 196, 228, 240, and 307 cm^−1^ for the gray lithology sliver (Figure 6b), combined with the fact that the sulfur content in the studied fragment increased in parallel with the increase in iron (Table 1—1st place for gray sliver), may indicate the presence of troilite (FeS) in this sliver [27]. Additionally, it is noteworthy that the Ribbeck meteorite contains sulfides rich in lithophile elements, which are typically removed during the leaching process. This could explain the sulfur smell at the meteorite fall site.

## 4. Conclusions

Raman spectroscopy was used to examine two different lithology fragments of the Ribbeck meteorite. The white lithology fragment showed a homogeneous chemical and phase structure characteristic of orthopyroxene, a mineral in the pyroxene family. This confirms that the Ribbeck meteorite is composed of about 76% FeO-free enstatite, a type of magnesium silicate mineral in the pyroxene family.

The gray lithology fragment of the Ribbeck meteorite was characterized by a greater diversity of chemical composition, which also translated into its greater phase diversity. In addition to the different contents of metals and silicon in the individual studied locations, the gray lithology fragment was also characterized by a lack of sodium, which occurred in the studied white fragment of the meteorite (average value of about 1.4 at.%). Moving on to the analysis of the phase composition of this fragment, the analysis of the Raman spectra showed that in addition to the pyroxenes characteristic of the white lithology fragment, minerals from the olivine group (fayalite and forsterite) could also be identified in it, as well as plagioclase (rather only anorthite contributed; there was a lack of sodium in the examined meteorite fragment) and sulfur, which was in the form of metal sulfides and its pure crystalline form. The EDS test results show that in the meteorite fragments there were areas with higher sulfur content, which was accompanied by higher iron content; respectively, the average value increased 5-fold to about 3.1 and 6-fold to 9.9 at.% in relation to the other tested locations.

## Figures and Tables

**Table 1:** Chemical composition of the tested samples obtained on the basis of EDS maps.

Atomic % of Element															
C	O	Na	Mg	Al	Si	S	Cl	K	Ca	Cr	Fe	Co	Ti	Ni	Mo
White sliver															
29.93	50.95	1.42	7.59	0.99	8.51	0.08	0.02	0.04	0.21	0.04	0.2	0.02	-	-	-
Gray sliver—1st place															
21.7	57.1	-	2.55	-	1.94	3.09	0.07	0.36	1.19	0.17	9.87	-	1.65	0.32	-
Gray sliver—2nd place															
30.5	53.94	-	8.61	-	6.61	-	-	-	0.09	0.06	0.09	0.05	-	-	0.06
Gray sliver—3rd place															
33.93	49.85	-	8.16	0.09	7.02	0.09	-	0.05	0.6	-	0.2	-	-	-	-

## Data Availability

The original contributions presented in the study are included in the article, further inquiries can be directed to the corresponding author.
